# Associations of Circadian Clock Gene Variants with Clinical Features and Treatment Characteristics in Ulcerative Colitis

**DOI:** 10.3390/jcm15083060

**Published:** 2026-04-17

**Authors:** Suleyman Yildirim, Fatima Ceren Tuncel, Celalettin Herek, Memduh Sahin, Sacide Pehlivan

**Affiliations:** 1 Department of Gastroenterology, Basaksehir Cam and Sakura City Hospital, University of Health Sciences, Istanbul 34480, Türkiye; celalettinherek@gmail.com (C.H.); memduh.sahin@sbu.edu.tr (M.S.); 2 Department of Medical Biology, Istanbul Faculty of Medicine, Istanbul University, Istanbul 34116, Türkiye; fatimaceren.tuncel@gmail.com (F.C.T.); sacide.pehlivan@istanbul.edu.tr (S.P.)

**Keywords:** BMAL1, CLOCK, CRY1, ulcerative colitis, circadian rhythm

## Abstract

**Background/Objectives**: Growing evidence suggests that disruption of circadian rhythms contributes to the pathogenesis of inflammation and inflammatory bowel disease; however, clinical data linking circadian gene variants to ulcerative colitis remain limited. In this study, we aimed to investigate associations between key circadian rhythm gene polymorphisms and clinical and treatment-related characteristics in ulcerative colitis. **Methods**: A total of 107 patients with ulcerative colitis and 80 healthy controls were included in this single-center cross-sectional study. The BMAL1 rs7950226, CLOCK rs1801260, and CRY1 rs2287161 polymorphisms were analyzed using the polymerase chain reaction–restriction fragment length polymorphism (PCR-RFLP) method. Genotype and allele frequency distributions were compared between patients and controls, and associations with clinical characteristics were evaluated within the ulcerative colitis cohort. **Results**: Genotype distributions of BMAL1 rs7950226 and CLOCK rs1801260 were similar between patients with ulcerative colitis and healthy controls; however, the G allele of BMAL1 was more frequent in patients (*p* = 0.028). Within the ulcerative colitis cohort, CLOCK rs1801260 genotypes were significantly associated with inflammatory and treatment-related characteristics, with the CC genotype linked to higher C-reactive protein levels (*p* = 0.021) and the TT genotype associated with increased azathioprine use (*p* = 0.006). **Conclusions**: These findings suggest a potential association between circadian rhythm gene variants and clinical features of ulcerative colitis, particularly in relation to inflammatory activity and treatment requirements, and provide preliminary clinical insight that warrants further investigation in larger and longitudinal studies.

## 1. Introduction

The circadian cycle is a rhythmic regulatory system in living organisms that organizes a wide range of physiological and behavioral activities in synchrony with the day–night cycle. Serving as an internal timekeeping system, it is present across humans, plants, fungi, and even some bacteria, and regulates key biological processes, including the sleep–wake cycle, hormone release, and cellular metabolism [1].

BMAL1 (Brain and Muscle ARNT-Like 1), CLOCK (Circadian Locomotor Output Cycles Kaput), PER1/2/3 (Period 1/2/3), and CRY1/2 (Cryptochrome 1/2) have been identified as the core regulatory genes of the circadian system [2]. The BMAL1 and CLOCK proteins form a heterodimeric complex that initiates the transcription of multiple clock-controlled genes, most notably PER and CRY. Following their accumulation in the cytoplasm, PER and CRY proteins translocate back into the nucleus, where they repress BMAL1/CLOCK activity and thereby inhibit their own transcription. As PER and CRY proteins undergo time-dependent degradation in the cytoplasm, this inhibitory effect is relieved, allowing transcriptional activity to resume. Through this tightly regulated process, a self-sustained feedback loop with approximately 24 h periodicity is established [2,3]. In humans, circadian control, in its most developed form, is centrally regulated by the suprachiasmatic nucleus in the hypothalamus, and disruption of this rhythmicity has been linked to a wide spectrum of pathological conditions, including psychiatric disorders, neurodegenerative diseases, metabolic disorders such as obesity and diabetes, as well as cancer development [4].

Accumulating evidence suggests that BMAL1 and CRY proteins are not only central regulators of circadian rhythmicity but also important modulators of inflammatory signaling [5]. BMAL1 has been shown to influence the production of IL-6 and TNF-α and to regulate STAT3 activation in colonic epithelial cells [6]. In contrast, CRY proteins interact with NF-κB–mediated transcriptional pathways, thereby suppressing pro-inflammatory cytokine release [7]. Disruption of circadian mechanisms has been shown, in animal models, to lead to increased cytokine secretion, impaired epithelial renewal, and increased severity of colitis [8,9]. In a recent study, epithelial-specific genetic deletion of BMAL1 in the intestinal epithelium was found to reduce colonic inflammation, underscoring the importance of the epithelial circadian clock in regulating intestinal immune responses [10].

In addition to the well-established links between circadian genes and inflammatory pathways, both epidemiological studies and experimental models have demonstrated that circadian rhythm and sleep disturbances interact with the development and disease course of inflammatory bowel disease (IBD). Nevertheless, studies specifically investigating BMAL1, CRY1 and CLOCK gene polymorphisms in IBD, as well as in other autoimmune and autoinflammatory disorders, remain scarce.

In this study, we aimed to compare circadian rhythm gene polymorphisms, including BMAL1 (rs7950226), CRY1 (rs2287161), and CLOCK (rs1801260), between patients with ulcerative colitis (UC) and healthy controls, and to evaluate their associations with clinical, endoscopic, and biochemical parameters.

## 2. Materials and Methods

This study was conducted between May 2024 and January 2025 at Basaksehir Cam and Sakura City Hospital, Istanbul. A total of 109 patients diagnosed with UC were initially enrolled. Two patients were later excluded after colonoscopic evaluation revealed ileal involvement, leaving 107 patients for the final analysis. Patients with malignancy, advanced heart failure, drug-induced colitis or indeterminate colitis were excluded. The control group consisted of age and sex-matched healthy individuals without known chronic diseases, not receiving regular medication, and without a reported family history of IBD.

For patients with UC, demographic characteristics, disease duration, and family history of IBD were recorded. Current medical treatments were categorized into four groups: (1) 5-aminosalicylic acid (5-ASA), (2) azathioprine (AZA), (3) biologic agents, and (4) small-molecule therapies (e.g., Janus kinase inhibitors such as upadacitinib). Treatment groups were defined according to the highest-level therapy, as patients could receive concomitant 5-ASA or AZA as add-on therapy.

Patients’ colonoscopic activity was determined from procedures performed either during routine surveillance or to evaluate disease activation. We assessed endoscopic disease activity using the Mayo Endoscopic Subscore (MES; 0 = normal/inactive, 1 = mild erythema with decreased vascular pattern, 2 = marked erythema with absent vascular pattern, friability, and erosions, 3 = spontaneous bleeding and ulceration) and the Rachmilewitz Endoscopic Activity Index (EAI; 0–12; granulation, vascular pattern, friability, and mucosal damage) [11,12]. Based on the extent of colonoscopic involvement, patients were categorized into four groups: proctitis, left-sided colitis, extensive colitis, and pancolitis. Clinical disease activity was evaluated using the Mayo Clinical Score, which includes stool frequency, rectal bleeding, physician’s global assessment, and endoscopic findings. The following laboratory parameters were also recorded: white blood cell count (WBC), hemoglobin (Hb), platelet count (PLT), and C-reactive protein (CRP).

### 2.1. Genetic Analyses and Genotyping

Leukocytes were isolated from EDTA-anticoagulated whole blood, and genomic DNA was extracted using a commercial kit (HiPure Blood DNA Mini Kit, Magen, Guangzhou, China). Polymorphisms in BMAL1 (rs7950226), CLOCK (rs1801260), and CRY1 (rs2287161) were analyzed using the polymerase chain reaction–restriction fragment length polymorphism (PCR–RFLP) method.

**Figure 1:**
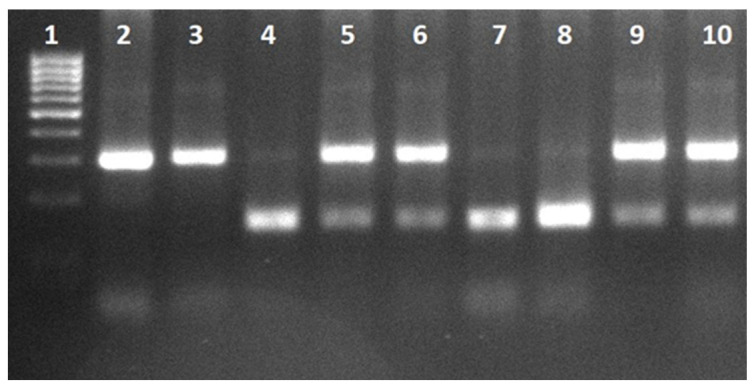
BMAL1 gene rs7950226 PCR and restriction digest products. Lane 1: DNA ladder (100–1000 bp); Lane 2: undigested PCR product (310 bp); Lane 3: GG (310 bp); Lanes 5, 6, 9, 10: GA (310, 159, 151 bp); Lanes 4, 7, 8: AA (159, 151 bp).

BMAL1 rs7950226. Primers: Forward 5′-CATGCTGTGCTTGAATACTCCT-3′, Reverse 5′-CTATGAAACCAAGGCTGAAACA-3′. PCR conditions: 95 °C for 5 min; 35 cycles of 95 °C for 30 s, 58 °C for 30 s, and 72 °C for 30 s; final extension at 72 °C for 5 min. Undigested amplicon size was 310 bp. PCR products were digested with RsaI at 37 °C for 24 h and fragments were resolved on 3% agarose gel at 110 V for 65 min. Genotypes: GG = 310 bp; GA = 310/159/151 bp; AA = 159/151 bp (Figure 1) [13].

**Figure 2:**
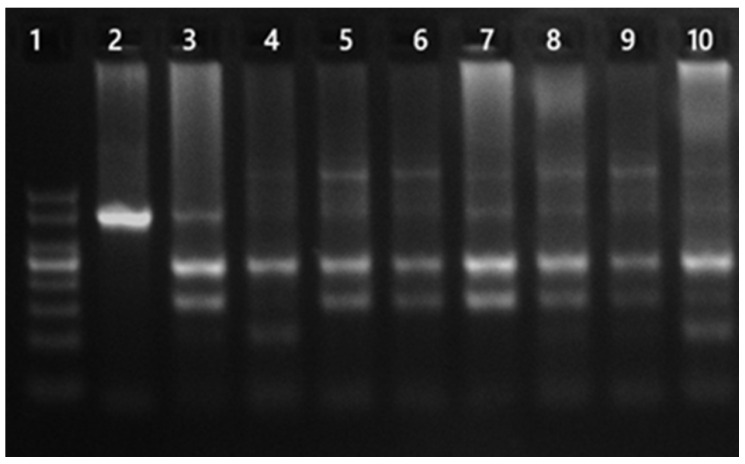
CRY1 gene rs2287161 PCR and restriction digest products. Lane 1: DNA ladder (50–500 bp); Lane 2: undigested PCR product (382 bp); Lanes 3,5,6,7,8,9: CC (226, 156 bp); Lanes 10: GC (226, 156, 108, 50 bp); Lane 4: GG (226, 108, 50 bp).

CRY1 rs2287161. Primers: Forward 5′-GGAACAGTGATTGGCTCTATCT-3′, Reverse 5′-GGTCCTCGGTCTCAAGAAG-3′. PCR conditions: 95 °C for 5 min; 35 cycles of 95 °C for 45 s, 58.4 °C for 45 s, and 72 °C for 45 s; final extension at 72 °C for 5 min. Undigested amplicon size was 382 bp. PCR products were digested with BseYI (37 °C for 60 min followed by 80 °C for 20 min) and fragments were resolved on 3% agarose gel at 110 V for 50 min. Genotypes: CC = 226/156 bp; GC = 226/156/108/50 bp; GG = 226/108/50 bp (Figure 2) [14].

**Figure 3:**
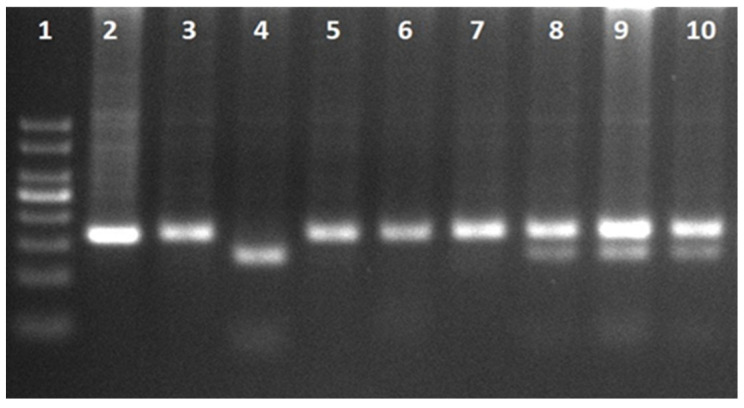
CLOCK gene rs1801260 PCR and restriction digest products. Lane 1: DNA ladder (50–500 bp); Lane 2: Undigested PCR product (167 bp); Lanes 3, 5, 6, 7: TT (167 bp); Lanes 8, 9, 10: TC (167, 129, 38 bp); Lane 4: CC (129, 38 bp).

CLOCK rs1801260. Primers: Forward 5′-GGGAAAGTTCCAGCAGTT-3′, Reverse 5′-ATCCAGGCACCTAAAACA-3′. PCR conditions: 95 °C for 5 min; 30 cycles of 95 °C for 30 s, 53 °C for 30 s, and 72 °C for 30 s; final extension at 72 °C for 5 min. Undigested amplicon size was 167 bp. PCR products were digested with SduI (Bsp1286I) at 37 °C for 24 h and fragments were resolved on 3% agarose gel at 110 V for 60 min. Genotypes: TT = 167 bp; TC = 167/129/38 bp; CC = 129/38 bp (Figure 3) [15].

### 2.2. Statistical Analysis

Statistical analyses were performed using IBM SPSS Statistics (version 25.0, IBM Corp., Armonk, NY, USA). Prior to analysis, the distribution of continuous variables was assessed with the Kolmogorov–Smirnov test. Given the non-normal distribution of variables, non-parametric tests were used for between group comparisons. Categorical data were analyzed using chi-square or Fisher’s exact tests. Multivariable logistic regression analysis was undertaken to assess factors independently associated with azathioprine use. Continuous data are presented as median (interquartile range) and categorical data as n (%). All statistical tests were two-sided and *p* < 0.05 was considered statistically significant.

To account for multiple testing, false discovery rate (FDR) correction was applied using the Benjamini–Hochberg method. FDR adjustment was restricted to predefined clinically relevant outcomes, including inflammation-related parameters (C-reactive protein levels and CRP positivity), treatment-related variables (azathioprine use), and representative disease activity measures. To avoid redundancy due to collinearity among endoscopic indices, only endoscopic remission was included in the FDR correction, while other correlated endoscopic scores were excluded. Non-specific laboratory parameters (e.g., white blood cell count, hemoglobin, platelet count) and descriptive variables were not included in the FDR adjustment. This study was prepared and reported in accordance with the STROBE guidelines.

## 3. Results

A total of 107 patients with UC (57.2%) and 80 healthy controls (42.8%) were included in the study. The mean age was 41.6 years in the UC group and 41.0 years in the control group. Females accounted for 56 patients (52.3%) in the UC group and 43 individuals (53.8%) in the control group. Age and sex did not differ significantly between the groups.

### 3.1. Association of Genotype Distributions with Clinical Parameters

Across all three circadian rhythm genes (BMAL1, CLOCK, and CRY1), no significant associations were observed between genotype distributions and demographic characteristics or disease-related variables, including age at diagnosis, disease duration, and disease extent. Furthermore, genotype distributions were not significantly associated with either endoscopic or clinical disease activity or remission status. However, CRP levels tended to be lower in patients carrying the BMAL1 GG genotype compared with those carrying the AA or GA genotypes, although this difference did not reach statistical significance (*p* = 0.06). When we categorized CRP levels according to the predefined clinical threshold (>5 mg/L), a significant association emerged between BMAL1 genotype and CRP positivity (*p* = 0.037) (Table 1).

As shown in Table 2, a significant association was observed between CLOCK genotypes and thioprine treatment (*p* = 0.022). When analyzed under a dominant C-allele model (CC + TC vs. TT), azathioprine use was considerably higher in TT carriers than in C-allele carriers (37.9% vs. 14.3%; χ^2^(1) = 7.52, *p* = 0.006; Fisher’s exact *p* = 0.008). TT carriers also showed approximately fourfold higher odds of azathioprine use compared with C-allele carriers in multivariable logistic regression analysis (adjusted OR = 4.1, 95% CI: 1.16–14.36; *p* = 0.009).

To address multiple testing, false discovery rate correction was applied using the Benjamini–Hochberg method. Following FDR adjustment, the association between CLOCK rs1801260 genotypes and azathioprine use remained statistically significant (FDR-adjusted *p* = 0.036), supporting the robustness of this finding. In contrast, associations involving CRP measures and BMAL1 genotype did not withstand correction for multiple comparisons.

No significant relationships were identified between CRY1 variants and the assessed clinical or laboratory characteristics (Table A1).

### 3.2. Genotype Distributions in Ulcerative Colitis and Control Group

The distribution of BMAL1 rs7950226 genotypes did not differ significantly between the UC group and controls (*p* = 0.10). However, allelic analysis revealed a significantly higher frequency of the G allele in patients with UC compared with controls (49.5% vs. 38.0%, *p* = 0.028). Analysis of the CLOCK rs1801260 variant revealed no significant differences in genotype frequencies between groups (*p* = 0.97).

For CRY1 rs2287161, genotype distributions differed significantly between groups (*p* = 0.002). The GC genotype was more frequent in patients with UC (42.1%) compared with controls (18.8%), whereas the CC genotype was less frequent among patients (43.9% vs. 57.5%) (Table A2). Hardy–Weinberg equilibrium analysis showed that genotype distributions in both the UC and control groups were consistent with equilibrium for BMAL1 rs7950226 and CLOCK rs1801260 (*p* > 0.05). In contrast, a significant deviation from equilibrium was observed for CRY1 rs2287161 in the control group (*p* < 0.001), while the UC group remained consistent with equilibrium.

## 4. Discussion

The circadian rhythm regulates the body’s daily physiological and behavioral rhythms by coordinating sleep patterns as well as hormonal, metabolic, and immune functions [16]. Disruption of this rhythm, such as that observed in individuals working rotating or night shifts, has been associated with an increased incidence of IBD and has been suggested to represent an independent risk factor for IBD development [17]. Beyond occupational factors, population-based data also indicate that fatigue and reduced sleep quality are highly prevalent among patients with IBD [18,19,20,21].

It is increasingly well recognized that the circadian rhythm regulates homeostasis in the intestinal mucosa by coordinating barrier integrity, absorption, and epithelial regeneration [22,23,24,25]. In an experimental mouse model with a disrupted sleep–wake cycle, increased intestinal permeability was observed, leading to the development of alcohol-induced hepatic inflammation [26]. Poroyko and colleagues demonstrated that chronic sleep disruption may lead to intestinal inflammation and metabolic dysfunction by altering the gut microbiota composition [27]. In parallel, multiple experimental studies have shown that circadian rhythm disruption can exacerbate colitis severity by increasing inflammatory responses through effects on the microbiota and core clock genes [8,28,29,30,31].

Taken together, both epidemiological and tissue-level findings indicate that circadian genes such as BMAL1, CRY, and CLOCK play a pivotal role in regulating inflammation, epithelial barrier integrity, and mucosal immune function, thereby providing a strong biological rationale for investigating functional genetic variants of these genes in patients with UC.

### 4.1. Clinical Parameters and Circadian Gene Variants

While the BMAL1 rs7950226 and CLOCK rs1801260 genotype distributions did not differ significantly between patients and controls, several associations with clinical and laboratory parameters were observed. In particular, patients carrying the BMAL1 GG genotype exhibited lower CRP levels compared with those carrying the AA and GA genotypes, and a significant association was identified when CRP was analyzed as a categorical variable. Similarly, in the CLOCK rs1801260 variant, patients with the CC genotype showed higher CRP levels than those with the TC or TT genotypes, with consistent findings in categorical analyses. However, these associations did not remain statistically significant after correction for multiple comparisons, indicating that they should be interpreted as exploratory findings. Nevertheless, the consistency of CRP-related trends across both BMAL1 and CLOCK variants may suggest a potential role of circadian genes in modulating inflammatory activity in ulcerative colitis. These observations may be interpreted in light of experimental studies demonstrating the influence of circadian clock components on inflammatory signaling pathways and mucosal barrier function.

Another key finding of our study was the association between CLOCK gene polymorphisms and azathioprine use. Notably, this association remained statistically significant after adjustment for multiple testing, supporting the robustness of this observation. Patients carrying the TT genotype exhibited a higher likelihood of azathioprine use, which may reflect a more treatment-resistant or fluctuating disease course requiring immunomodulatory escalation.

Although our finding should be interpreted with caution, as it is derived from a single polymorphism analysis, it provides an important clinical signal. It raises the possibility that circadian gene variants may influence not only inflammatory activity but also treatment requirements in ulcerative colitis. Future studies involving larger, independent cohorts are needed to confirm these findings and to further elucidate the underlying biological mechanisms.

At first glance, the observation that higher CRP levels were associated with the CC genotype, whereas azathioprine use and treatment escalation were more frequent in the TT and TC genotypes, may appear contradictory. However, CRP represents a cross-sectional marker of inflammatory burden, whereas the need for azathioprine reflects the cumulative clinical course, including steroid dependence, relapse frequency, and difficulty in maintaining remission. It is therefore plausible that CLOCK variants may influence not only the magnitude of inflammation but also its temporal dynamics, regulation, and response to therapy. In this context, TT genotypes may be associated with a more fluctuating or treatment-resistant disease course that necessitates immunomodulatory escalation despite lower contemporaneous CRP levels. This interpretation is further supported by the fact that 5-ASA has relatively limited systemic anti-inflammatory effects compared with immunomodulators and biologic agents and is therefore primarily used in patients with mild to moderate disease activity [32]. Consistently, the literature has frequently reported that CRP is an unreliable sole predictor in patients treated with azathioprine or biologics; particularly in UC, CRP sensitivity is limited, and combined assessment with fecal calprotectin and drug-level or anti-drug antibody measurements provides more robust predictive value [33,34].

To our knowledge, this study represents one of the first clinical investigations to directly evaluate circadian gene variants in patients with ulcerative colitis, thereby supporting a link between circadian rhythm genes and inflammation and addressing an important gap in the literature. Exploratory findings related to CRY1 should be interpreted with caution due to the deviation from Hardy–Weinberg equilibrium. In contrast, the clinically relevant findings observed for BMAL1 and CLOCK variants provide supporting evidence for a potential link between circadian rhythm pathways and clinical features of ulcerative colitis, thereby extending observations from experimental models to patient populations.

This study has several limitations. Although the cohort size was sufficient for the primary aims, it may have limited the sensitivity to detect more subtle associations. In addition, colonic tissue analyses were not performed, which might have provided complementary epigenetic insights beyond peripheral genotyping. We also did not include objective or questionnaire-based assessments of sleep patterns or circadian disruption, which could have added valuable phenotypic context; however, these evaluations were beyond the scope of the present work. Future studies with larger and more diverse patient populations, ideally integrating genetic, epigenetic, and circadian phenotyping data, will be essential to further clarify the role of circadian biology in IBD.

### 4.2. Circadian Gene Variants and Susceptibility Patterns in Ulcerative Colitis

In our study, the GC genotype of CRY1 rs2287161 was more frequently observed among patients with UC, whereas the GG and CC genotypes were more common in the control group. This pattern may suggest a potential difference related to the heterozygous genotype rather than a clear dominant or recessive effect. However, given the marked deviation from Hardy–Weinberg equilibrium observed in the control group for this polymorphism, these findings should be interpreted with caution and considered exploratory.

CRY1 has been implicated in the regulation of circadian rhythms and inflammatory responses. Experimental studies suggest that CRY proteins may modulate NF-κB–mediated inflammatory signaling, and disruption of this pathway has been associated with increased cytokine production [7]. While this biological background may support a potential link between circadian gene variants and inflammatory processes, the present findings do not allow definitive conclusions and require validation in larger studies using alternative genotyping approaches.

Allelic analysis demonstrated a higher frequency of the G allele of the BMAL1 rs7950226 polymorphism among patients with UC, despite the absence of a significant difference in genotype distributions. This finding may indicate a modest allelic effect that is not fully captured at the genotype level, potentially due to sample size limitations or genotype distribution patterns. In light of previous studies on the epithelial and inflammatory roles of BMAL1, this observation may provide a basis for future investigations into its potential role in IBD.

## 5. Conclusions

This study provides clinical insight into the potential role of circadian rhythm gene variants in ulcerative colitis, particularly in relation to inflammatory activity and treatment-related characteristics. Our findings may suggest that circadian rhythm genes deserve broader attention in immune-mediated inflammatory diseases, particularly Crohn’s disease, as well as other autoimmune and autoinflammatory conditions associated with chronic inflammation. A broader exploration of circadian biology and its underlying mechanisms across these disorders may help to better understand shared disease pathways, guide future research, and inform the development of potential treatment strategies and therapeutic targets.

## Figures and Tables

**Table 1:** Clinical and laboratory characteristics by *BMAL1* genotype in UC.

*BMAL1*	AA (*n* = 29)	GA (*n* = 50)	GG (*n* = 28)	*p*
Age (years) *	38.0 (19)	41.5 (27)	42.5 (24)	0.240 ^c^
**Sex**				
Male	16 (55.2)	23 (46)	12 (42.9)	0.617 ^a^
Female	13 (44.8)	27 (54)	16 (57.1)	
Age at diagnosis (years) *	27.0 (18)	33.5 (19)	34.0 (22)	0.199 ^c^
UC duration *	48 (104)	64 (109)	37.5 (93)	0.076 ^c^
**UC involvement**				
Proctitis	6 (20.7)	11 (22)	3 (10.7)	
Left-sided colitis	8 (27.6)	18 (36)	10 (35.7)	0.446 ^a^
Extensive colitis	7 (24.1)	16 (32)	10 (35.7)	
Pancolitis	8 (27.6)	5 (10)	5 (17.9)	
Rachmilewitz score *	7.0 (8)	6.0 (7)	7.0 (9)	0.579 ^c^
**Endoscopic remission**				
No	22 (75.9)	37 (74)	22 (78.6)	0.903 ^a^
Yes	7 (24.1)	13 (26)	6 (21.4)	
**Clinical remission**				
No	11 (37.9)	17 (34)	9 (32.1)	0.894 ^a^
Yes	18 (62.1)	33 (66)	19 (67.9)	
**5-aminosalicylic acid treatment**				
No	7 (24.1)	9 (18)	3 (10.7)	0.414 ^a^
Yes	22 (75.9)	41 (82)	25 (89.3)	
**Azathioprine treatment**				
No	21 (72.4)	40 (80)	17 (60.7)	0.184 ^a^
Yes	8 (27.6)	10 (20)	11 (39.3)	
**Anti-TNF treatment**				
No	18 (62.1)	39 (78)	22 (78.6)	0.240 ^a^
Yes	11 (37.9)	11 (22)	6 (21.4)	
**Other biologics or JAK inhibitors**				
No	25 (86.2)	42 (84)	27 (96.4)	0.283 ^a^
Yes	4 (13.8)	8 (16)	1 (3.6)	
**Treatment stage**				
Only 5-ASA	12 (41.4)	24 (48)	13 (46.4)	
Azathioprine ± 5-ASA	3 (10.3)	7 (14)	7 (25)	0.403 ^b^
Anti-TNF ± Azathioprine ± 5-ASA	11 (37.9)	11 (22)	7 (25)	
Other biologics or JAK inhibitors	3 (10.3)	8 (16)	1 (3.6)	
WBC * (×10^3^/µL)	6980 (2880)	7030 (2445)	6350 (2623)	0.238 ^c^
Hb * (g/dL)	13 (5)	12.5 (3)	12.8 (2)	0.929 ^c^
PLT * (×10^3^/µL)	319 (119)	267.5 (183.5)	269.5 (65)	0.745 ^c^
CRP * (mg/L)	3 (3)	3.5 (7.5)	1.5 (3.1)	0.060 ^c^
**CRP ≤ 5 mg/L**	23 (79.3)	29 (58)	23 (82.1)	**0.037 ^a^**
**CRP > 5 mg/L**	6 (20.7)	21 (42)	5 (17.9)	

5-ASA: 5-aminosalicylic acid; AZA: azathioprine; WBC: white blood cell count; Hb: hemoglobin; PLT: platelet count; CRP: C-reactive protein. Continuous variables (*) are presented as median (interquartile range), and categorical variables as n (%). ^a^ Chi-square test; ^b^ Fisher’s exact test; ^c^ Kruskal–Wallis test.

**Table 2:** Clinical and laboratory characteristics by *CLOCK* genotype in ulcerative colitis.

*CLOCK*	CC (*n* = 10)	TC (*n* = 39)	TT (*n* = 58)	*p*
Age (years) *	37 (13)	41 (18)	38 (21)	0.104 ^c^
**Sex**				
Male	4 (40)	19 (48.7)	28 (48.3)	0.877 ^a^
Female	6 (60)	20 (51.3)	30 (51.7)	
Age at diagnosis (years) *	26 (16.5)	35 (19)	33 (21.25)	0.223 ^c^
Disease duration (months) *	54 (103)	70 (126)	46 (93)	0.191 ^c^
**UC involvement**				
Proctitis	1 (10)	9 (23.1)	10 (17.2)	
Left-sided colitis	3 (30)	13 (33.3)	20 (34.5)	0.215 ^b^
Extensive colitis	6 (60)	13 (33.3)	14 (24.1)	
Pancolitis	0 (0)	4 (10.3)	14 (24.1)	
Rachmilewitz score *	7 (4.5)	7 (10)	7 (7.25)	0.707 ^c^
**Endoscopic remission**				
No	8 (80)	28 (71.8)	45 (77.6)	0.765 ^a^
Yes	2 (20)	11 (28.2)	13 (22.4)	
**Clinical remission**				
No	5 (50)	16 (41)	16 (27.6)	0.221 ^a^
Yes	5 (50)	23 (59)	42 (72.4)	
**5-ASA treatment**				
No	1 (10)	7 (17.9)	11 (19)	0.790 ^a^
Yes	9 (90)	32 (82.1)	47 (81)	
**Azathioprine treatment**				
No	9 (90)	33 (84.6)	36 (62.1)	**0.022 ^a^**
Yes	1 (10)	6 (15.4)	22 (37.9)	
**Anti-TNF treatment**				
No	8 (80)	28 (71.8)	43 (74.1)	0.868 ^a^
Yes	2 (20)	11 (28.2)	15 (25.9)	
**Other biologics or JAK inhibitors**				
No	9 (90)	36 (92.3)	49 (84.5)	0.510 ^b^
Yes	1 (10)	3 (7.7)	9 (15.5)	
**Treatment stage**				
Only 5-ASA	6 (60)	21 (53.8)	22 (37.9)	
Azathioprine ± 5-ASA	1 (10)	4 (10.3)	12 (20.7)	0.632 ^b^
Anti-TNF ± Azathioprine ± 5-ASA	2 (20)	11 (28.2)	16 (27.6)	
Other biologics or JAK inhibitors	1 (10)	3 (7.7)	8 (13.8)	
WBC * (×10^3^/µL)	7475 (1992.5)	7470 (2273)	6705 (2815)	0.137 ^c^
Hb * (g/dL)	11.65 (2.47)	13 (2.3)	12.7 (3.7)	0.076 ^c^
PLT * (×10^3^/µL)	306 (209)	285 (82)	287 (135.5)	0.823 ^c^
CRP * (mg/L)	9.1 (19.93)	2.5 (6.4)	2.4 (4.05)	**0.021 ^c^**
**CRP ≤ 5 mg/L**	3 (30)	28 (71.8)	44 (75.9)	**0.013 ^a^**
**CRP > 5 mg/L**	7 (70)	11 (28.2)	14 (24.1)	

5-ASA: 5-aminosalicylic acid; AZA: azathioprine; WBC: white blood cell count; Hb: hemoglobin; PLT: platelet count; CRP: C-reactive protein. Continuous variables (*) are presented as median (interquartile range), and categorical variables as n (%). ^a^ Chi-square test; ^b^ Fisher’s exact test; ^c^ Kruskal–Wallis test.

## Data Availability

The data presented in this study are available on reasonable request from the corresponding author.

## Abbreviations

The following abbreviations are used in this manuscript:

IBD	Inflammatory bowel disease
UC	Ulcerative colitis
AZA	Azathioprine
5-ASA	5-aminosalicylic acid

## Appendix A

**Table A1:** Clinical and laboratory characteristics by *CRY1* genotype in UC.

CRY1	CC (*n* = 47)	GC (*n* = 45)	GG (*n* = 15)	*p*
**Age (years) ***	45 (22)	39 (21)	39 (15)	0.167 ^c^
**Sex**				
Male	23 (48.9)	22 (48.9)	6 (40)	0.814 ^a^
Female	24 (51.1)	23 (51.1)	9 (60)	
**Age at diagnosis (years) ***	39 (20)	31 (20)	29 (20)	0.230 ^c^
**Disease duration (months) ***	72 (100)	39 (105)	38 (42)	0.083 ^c^
**UC involvement**				
Proctitis	6 (12.8)	10 (22.2)	4 (26.7)	
Left-sided colitis	18 (38.3)	11 (24.4)	7 (46.7)	0.290 ^b^
Extensive colitis	17 (36.2)	14 (31.1)	2 (13.3)	
Pancolitis	6 (12.8)	10 (22.2)	2 (13.3)	
**Rachmilewitz score ***	6 (6)	7 (9)	7 (8)	0.647 ^c^
**Endoscopic remission**				
No	36 (76.6)	34 (75.6)	11 (73.3)	0.967 ^a^
Yes	11 (23.4)	11 (24.4)	4 (26.7)	
**Clinical remission**				
No	18 (38.3)	14 (31.1)	5 (33.3)	0.765 ^a^
Yes	29 (61.7)	31 (68.9)	10 (66.7)	
**5-ASA treatment**				
No	5 (10.6)	11 (24.4)	3 (20)	0.216 ^a^
Yes	42 (89.4)	34 (75.6)	12 (80)	
**Azathioprine treatment**				
No	34 (72.3)	34 (75.6)	10 (66.7)	0.793 ^a^
Yes	13 (27.7)	11 (24.4)	5 (33.3)	
**Anti-TNF treatment**				
No	34 (72.3)	35 (77.8)	10 (66.7)	0.665 ^a^
Yes	13 (27.7)	10 (22.2)	5 (33.3)	
**Other biologics or JAK inhibitors**				
No	41 (87.2)	39 (86.7)	14 (93.3)	0.779 ^a^
Yes	6 (12.8)	6 (13.3)	1 (6.7)	
**Treatment stage**				
Only 5-ASA	21 (44.7)	21 (46.7)	7 (46.7)	
Azathioprine ± 5-ASA	7 (14.9)	7 (15.6)	3 (20)	0.883 ^b^
Anti-TNF ± Azathioprine ± 5-ASA	13 (27.7)	11 (24.4)	5 (33.3)	
Other biologics or JAK inhibitors	6 (12.8)	6 (13.3)	0 (0)	
**WBC *** (×10^3^/µL)	6400 (2670)	7300 (2182)	6770 (3610)	0.183 ^c^
**Hb *** (g/dL)	12.8 (3)	12.9 (3)	12.6 (4)	0.786 ^c^
**PLT *** (×10^3^/µL)	286 (127)	270 (133)	317 (94)	0.311 ^c^
**CRP *** (mg/L)	3.1 (5.5)	2.1 (4.5)	2.4 (6.8)	0.531 ^c^

5-ASA: 5-aminosalicylic acid; AZA: azathioprine; WBC: white blood cell count; Hb: hemoglobin; PLT: platelet count; CRP: C-reactive protein. Continuous variables (*) are presented as median (interquartile range), and categorical variables as n (%). ^a^ Chi-square test; ^b^ Fisher’s exact test; ^c^ Kruskal–Wallis test.

**Table A2:** Genotype distribution of circadian genes in UC and controls.

*BMAL1* Genotypes (rs7950226)	Ulcerative Colitis N (%) n:107	Control N (%) n:80	*p*
GG	28 (26.2%)	14 (17.5%)	0.1
GA	50 (46.7%)	33 (41.3%)	
AA	29 (27.1%)	33 (41.3%)	
G	106 (49.5%)	61 (38.1%)	**0.028**
A	108 (50.5%)	99 (61.9%)	
***CLOCK*** **Genotypes (rs1801260)**			
TT	58 (54.2%)	42 (52.5%)	0.971
TC	39 (36.5%)	30 (37.5%)	
CC	10 (9.3%)	8 (10%)	
T	155 (72.4%)	114 (71.3%)	0.802
C	59 (27.6%)	46 (28.8%)	
***CRY1*** **Genotypes (rs2287161)**			
GG	15 (14%)	19 (23.7%)	**0.002 ***
GC	45 (42.1%)	15 (18.8%)	
CC	47 (43.9%)	46 (57.5%)	
G	75 (35%)	53 (33.1%)	0.698
C	139 (65%)	107 (66.9%)	

* CRY1 rs2287161 results should be interpreted with caution due to deviation from Hardy–Weinberg equilibrium in the control group.
